# Understanding immunosuppression medication adherence in kidney transplant recipients: a cross-sectional exploration of the role of illness perceptions, medication beliefs and perceived behavioural control

**DOI:** 10.1080/21642850.2025.2562857

**Published:** 2025-10-14

**Authors:** Rosie Heape, Antonia J. Cronin, Lyndsay Hughes

**Affiliations:** a School of Immunology & Microbial Sciences, Faculty of Life Sciences and Medicine, King’s College London, London, UK; b Centre for Nephrology, Urology and Transplantation, King's College London, London, UK; c Department of Nephrology and Transplantation, Guy's and St Thomas' NHS Trust, London, UK; d Psychology Department, Institute of Psychiatry, Psychology and Neuroscience, King’s College London, London, UK

**Keywords:** Medication adherence, immunosuppression, kidney transplant, common-sense model, perceived behavioural control

## Abstract

**Objective:**

Non-adherence to immunosuppression medication (ISM) is common in kidney transplant recipients (KTRs), despite being associated with poor clinical outcomes. Understanding potentially modifiable contributors to non-adherence is essential for developing effective interventions. This study explored the relationship between components of the Common-Sense Model (CSM), including illness perceptions (graft-specific and of kidney disease more broadly) and beliefs about ISM, as well as perceived behavioural control (PBC), and total, intentional and unintentional ISM non-adherence in KTRs.

**Methods and measures:**

A cross-sectional observational study was conducted with *N* = 296 KTRs. Participants completed self-report measures including the Brief Illness Perception Questionnaire, Beliefs about Medicines Questionnaire, questions assessing PBC, and the Medication Adherence Report Scale. Hierarchical binary logistic regression analyses were conducted to examine the explanatory value of variables on adherence outcomes.

**Results:**

Over half of participants (57%) reported any indication of non-adherence. Unintentional non-adherence was reported more frequently (54%) than intentional non-adherence (14%). Combining CSM components with PBC best explained variance in total (Nagelkerke *R*^2^ = 19.8%), intentional (Nagelkerke *R*^2^ = 15.5%), and unintentional non-adherence (Nagelkerke *R*^2^ = 19.3%).

**Conclusion:**

Enhancing PBC around taking ISM may offer a valuable intervention target, particularly when addressed alongside CSM components to reduce both intentional and unintentional non-adherence.

## Introduction

Chronic kidney disease (CKD) is a long-term condition characterised by progressive and irreversible decline in kidney function. End-stage kidney disease (ESKD), or CKD stage five, occurs when function declines to less than 15% of normal capacity (NHS Blood and Transplant, 2025). ESKD is ultimately fatal and requires renal replacement therapy with either dialysis or kidney transplantation. Transplantation is the preferred treatment, offering better quality of life and survival than dialysis (Laupacis et al., 1996; Wolfe et al., 1999). Lifelong immunosuppression (anti-rejection) medication (ISM) is essential for kidney transplant recipients (KTRs), typically involving triple immunosuppressive therapy consisting of a calcineurin inhibitor, an antiproliferative agent and a corticosteroid. Taking ISM as prescribed (termed ‘adherence’) is crucial to reduce the risk of poor outcomes such as graft rejection, failure and loss. Missing as little as 5% of prescribed doses has been associated with significantly greater acute rejection and graft loss (Nevins et al., 2001; Nevins & Thomas, 2009). Moreover, the odds of graft failure increase sevenfold in non-adherent patients compared with adherent patients (Butler et al., 2004). Consistent with EMERGE guidelines (De Geest et al., 2018), the present study focused on the implementation phase of ISM adherence, reflecting the importance of daily, lifelong adherence in KTRs.

Despite the importance of adhering to ISM, non-adherence in KTR is common at 36%−55% (Gokoel et al., 2020). Research has identified demographic correlates, such as younger age, male sex and greater time since transplantation (Corr et al., 2024), and non-specific psychosocial factors such as depression and work and social functioning (Melilli et al., 2025). However, these factors offer little insight into underlying motivators and are not modifiable through interventions. To develop effective interventions, it is necessary to distinguish between *intentional* non-adherence, where a decision is made not to take medication as prescribed, and *unintentional* non-adherence, which may be due to forgetting to take medication or not understanding the prescribed instructions. Research in long-term conditions has shown that these behaviours are driven by different underlying factors that may require different methods to address (De Vries et al., 2014; Hope et al., 2020; Moon et al., 2017). Intentional non-adherence is typically linked to perceptual barriers, whereas unintentional non-adherence is more often associated with practical barriers (Horne et al., 2005). The Common-Sense Model of Self-Regulation (CSM) (Leventhal et al., 2012) provides a theoretical framework for understanding behavioural mechanisms driving treatment adherence, which prior studies have shown to be modifiable through intervention (Moon et al., 2019; Perera et al., 2014; Petrie et al., 2012). The CSM proposes that beliefs formed about the perceived causes, consequences, timeline, coherence, emotional response and personal and treatment control of an illness directly influence coping responses to health threats, such as kidney graft rejection or failure. The necessity-concerns framework (Horne & Weinman, 2002), an extension to the CSM, proposes that adherence is influenced by a judgement of personal need for the treatment (necessity beliefs) and concerns surrounding the treatment.

Illness and treatment beliefs have consistently been linked to adherence in long-term conditions, including CKD (Broadbent et al., 2011; Chilcot et al., 2010; Horne & Weinman, 2002; Horne et al., 2013; Wileman et al., 2015). However, evidence in KTRs remains limited and inconsistent. Some studies report stronger necessity beliefs and fewer concerns to be associated with better ISM adherence (Griva et al., 2012), whereas others show no association (Lennerling & Forsberg, 2012; Massey et al., 2013). Evidence for illness perceptions is similarly inconsistent (Cossart et al., 2019; Massey et al., 2015; Wang et al., 2022).

Importantly, KTRs face a dual health threat: the risk of graft loss or failure *and* the continued experience of living with CKD even after transplantation. Previous studies have examined *either* transplant graft-related perceptions *or* perceptions related to living with CKD more broadly (Cossart et al., 2019; Massey et al., 2013; Wang et al., 2022).

The dual health threat is complicated by the fact that graft rejection can occur despite full ISM adherence, potentially leading some KTRs to question the value of strict adherence. Therefore, understanding KTR’s perceptions of both their kidney transplant (hereafter referred to as graft-related perceptions) and their continued experience of living with CKD post-transplant (hereafter referred to as CKD perceptions) is important. Exploring how each relates to medication taking will provide a more nuanced understanding of medication self-management in this complex population.

Given the limited variance in adherence explained by CSM constructs alone, additional factors likely play a role. The Theory of Planned Behaviour (Azjen, 1991), another widely applied model to explain health behaviours, may offer complimentary insights. According to the TPB, behaviour such as medication adherence is driven by three components: attitudes towards the behaviour, subjective norms and perceived behavioural control (PBC) i.e. one's perceived control over engaging in the behaviour. PBC is particularly relevant to KTRs, who take on average 11.6 pills daily (Oosting et al., 2024), and of whom half have at least one comorbidity (Wu et al., 2020). A high pill burden increases the complexity of medication management, making PBC important as those with higher PBC may be more likely to establish effective strategies to adhere to a demanding regimen. PBC has predicted intention to adhere to ISM over and above other TPB components (Chisholm et al., 2007; Yang et al., 2022) and was shown to be the strongest predictor to adherence behaviour in long-term conditions, with a significantly larger effect than attitudes and subjective norms (Rich et al., 2015).

Exploring the CSM alongside the PBC component of TPB may therefore provide a better explanation of adherence. The CSM captures treatment and personal control, often extending to broader self-management, whereas PBC captures actual control over medication taking. CSM perceptions also clarify why individuals hold certain illness and treatment beliefs, offering more specific insight than the TPB attitudes component, which only reflects a positive or negative evaluation of taking medication.

Combining TPB and CSM variables best explained intentional non-adherence to treatment in breast cancer survivors, with higher PBC (OR = 0.37, 95% CI = 0.24−0.56) linked to lower odds of non-adherence (Moon et al., 2017). Similarly, Orbell et al. (2006) found improved prediction of cervical screening attendance when both models were combined. Integrating PBC with CSM variables may therefore offer a more complete understanding of adherence behaviours, and more specific intervention targets.

The combined role of PBC with CSM variables has yet to be applied to medication adherence in KTRs, and key psychological drivers remain poorly understood. Few studies distinguish between intentional and unintentional behaviours, and the role of graft- and CKD-related perceptions remains underexplored. To address this gap, the present study aimed to examine the relationship between CSM components, (graft- and CKD-related perceptions, beliefs about ISM), with the addition of PBC around medication taking, and total, intentional and unintentional non-adherence. Consistent with previous research (Moon et al., 2017), we sought to better understand different types of adherence behaviour by identifying correlates of both intentional and unintentional non-adherence. By testing CSM variables alongside PBC, we aimed to identify distinct determinants of adherence behaviours and evaluate whether integrating models improves explanatory power, ultimately offering insight into targets for intervention.

We hypothesised that:

1. Unintentional non-adherence would be more frequently reported than intentional non-adherence.
2. Clinical and demographic variables, such as greater pill burden and depression would be significantly associated will greater general non-adherence.
3. Unfavourable CKD and graft-related perceptions (e.g. lower personal control, lower coherence), lower necessity beliefs, higher concerns, and lower perceived behavioural control (PBC) would be significantly associated with greater intentional non-adherence.
4. The addition of PBC to CSM variables would increase the variance explained in all adherence behaviours.

## Methods

### Design

This cross-sectional observational study recruited kidney patients at Guy’s Hospital Kidney Transplant Clinic in London, UK between 21/09/2022 and 03/10/2023. The study was conducted under the existing overarching ethical approval for Integrating Mental & Physical Healthcare: Research, Training & Services (IMPARTS) data to be used for research purposes (South Central – Oxford C REC: 23/SC/0109).

### Sample size

An a priori sample size calculation was conducted for logistic regression, informed by effect size estimates reported in Wang et al. (2022). Assuming a medium effect size (Nagelkerke’s R² = 0.09), an alpha of 0.05, power of 0.80, and 20 explanatory variables, the required sample size was estimated to be 249 participants. As the final set of explanatory variables for inclusion in the logistic regression models had not yet been determined, the calculation was conservatively based on a maximum of 20 predictors.

### Participants and procedure

Patients were eligible if they were 18 or over, living with a kidney transplant, prescribed immunosuppression medication and could read or speak English. Patients attending their routine outpatient appointment were approached by a researcher in the waiting room where informed consent was taken. Participants completed a 20−40 minute iPad-based questionnaire, consisting of IMPARTS self-report measures. Responses were automatically uploaded to electronic patient records (EPR) on completion. Demographic and clinical data were extracted from EPR by a data manager (KC), a clinician (AJC) and a researcher (RH).

## Measures

### Sociodemographic and clinical variables

Demographic and clinical data included age, sex, ethnicity (White/minority ethnic), time since transplant, donor type (living/deceased), number of kidney transplants (1/>1), number of prescribed ISM(s), number of tablets prescribed per day (all medications), comorbidity which was evaluated using the Charlson Comorbidity Index (CCI) score, where higher scores indicate greater morbidity (Charlson et al., 1987), and clinical diagnosis of depression or anxiety.

Index of multiple deprivation (IMD) quintile was used as a proxy for socioeconomic status, calculated using the English Indices of Deprivation 2019 Postcode Lookup based on postcode extracted from EPR (Ministry of Housing, Communities & Local Government, opendatacommunities.org, 2019). Quintiles ranged from most deprived (quintile 1) to least deprived (quintile 5).

Clinical test results collected on the day of screening ( + /− 14 d) were extracted, including serum creatinine (μmol/L) and estimated glomerular filtration rate (eGFR, ml/min/1.73²). Creatinine is a waste product formed during muscle metabolism; elevated levels may indicate impaired kidney function. Estimated GFR reflects how well the kidneys filter waste and excess water, with lower values indicating worse kidney function.

### Psychosocial variables

Distress was measured using the Patient Health Questionnaire−4 for Depression and Anxiety (PHQ−4) (Kroenke et al., 2009), a brief screening tool shown to be reliable and valid in clinical settings. Items are rated on a four-point scale from 0 (not at all) to 3 (nearly every day), with total scores ranging 0−12.

The Work and Social Adjustment Scale (WSAS) (Mundt et al., 2002) is a five-item measure of functional impairment due to kidney disease. Scores range 0−40 with higher scores indicating higher levels of impairment.

Beliefs about immunosuppression medication were assessed using the BMQ-Specific (Horne et al., 1999). Two five-item subscales assess beliefs about the necessity of, and concerns surrounding medication. Item wording was adapted to fit the context of ISM. Subscale total scores range 5−25. A necessity-concerns (*N*-C) differential was calculated by subtracting the total concerns score from the total necessity beliefs score. A positive differential suggests concerns are outweighed by necessity beliefs.

The Brief Illness Perception Questionnaire (BIPQ) (Broadbent et al., 2006) measured perceptions about kidney disease and, separately, perceived risk of graft failure. To measure both concepts, the BIPQ was administered twice, each time as a distinct questionnaire with adapted wording. Each adaptation included items rated on a 0−10 scale: six graft-related items and eight CKD-related items. Items measured cognitive representations (including perceived consequences, timeline, personal control, treatment control, identity and coherence) as well as emotional representations (concerns and emotional response).

To illustrate, coherence in the context of CKD captured an individual’s perceived understanding of their condition (*‘How well do you feel you understand your condition’*). In the context of risk of graft failure, coherence reflected an individual’s perceived understanding of their risk of graft failure (*‘How well do you feel you understand your risk of kidney transplant failure’*). Similarly, personal control over kidney disease was measured by asking *‘How much control do you feel you have over your condition?’*, while the graft-specific version asked *‘How much control do you feel you have over your risk of transplant failure?’.* The BIPQ has previously demonstrated good test-retest reliability and concurrent validity with the Illness Perception Questionnaire-Revised (Moss-Morris et al., 2002) among renal patients (Broadbent et al., 2006).

Perceived behavioural control surrounding medication taking was measured using four items with a 7-point response scale, developed following guidelines from Francis et al. (2004) and Ajzen (2006). Consistent with guidelines, two items assessed self-efficacy, e.g. *‘I am confident that I can take my kidney medication daily’*, and two items assessed controllability, e.g. *‘I feel in control of whether I take my kidney medication daily’*. Total scores ranged 4−28, with lower scores indicating poorer PBC. PBC differs from treatment and personal control in that it captures perceived control specifically over the behaviour of taking ISM, whereas personal and treatment control reflect broader perceptions involving other self-management behaviours.

The Medication Adherence Report Scale−5 (MARS; Horne & Weinman, 2002) measured self-reported adherence to ISM, scored on a five-point scale of never-always. One item assesses unintentional (forgetting) non-adherent behaviour (range 1−5; *‘I forget to take these medicines’*). Four items assess intentional non-adherent behaviour (range 4−20; *‘I alter the dose’*, *‘I stop taking them for a while’*, *‘I decide to miss out doses’*, *‘I take less than instructed’*). Higher scores indicate better adherence.

In line with previous literature (de Vries et al., 2014; Griva et al., 2018; Molloy et al., 2014), participants were classed as overall non-adherent if they scored ≤ 24, unintentionally non-adherent if they scored ≤ 4 and intentionally non-adherent if they scored ≤ 19 on the respective subscales; i.e. if they demonstrated any level of non-adherence when responding to these questions. Participants could be classed as both intentionally and unintentionally non-adherent. Dichotomising self-reported adherence is recommended when MARS scores are strongly skewed (de Vries et al., 2014), as was the case with these data.

## Statistical analysis

All data were analysed using SPSS v29 (IBM Corp, 2023). Descriptive statistics were used to summarise sample characteristics and to compare the frequency of intentional and unintentional non-adherence. Differences in demographic and clinical characteristics between adherent and non-adherent participants were examined using t-tests, Mann–Whitney U tests or χ2 as appropriate, with a Bonferroni-adjusted *p*-value of *p* ≤ .001 to correct for multiple testing. Pearson’s correlation tested for associations between illness and graft-related perceptions, treatment beliefs, PBC and continuous adherence scores. Adherence scores were treated as continuous here to preserve variance and statistical power. A *p-*value of <.05 was considered statistically significant.

Three hierarchical binary logistic regressions were constructed to assess whether the addition of PBC to CSM variables improved the variance explained in total, intentional and unintentional non-adherence. Models followed a data-driven approach, by which variables that were statistically significant in bivariate analyses were added in blocks: (1) demographic and clinical variables; (2) distress, diagnosis of depression/anxiety and work and social functioning; (3) CSM components and PBC.

Nagelkerke's R² was used to determine the ability of variables to explain non-adherence. Model fit was assessed by the −2 Log Likelihood statistic (−2LL). Odds ratios (OR) and 95% confidence intervals are reported to illustrate the findings.

Data were examined to assess the extent of missingness. Data were missing completely at random as demonstrated by Little’s MCAR test, χ2 (14) = 19.125, *p* = .160. Multiple imputation was conducted for total scores of the PHQ−4 (*N* = 20, 7% missing), WSAS (*N* = 23, 8% missing), BIPQ (CKD-related perceptions) (*N* = 41, 14% missing) and PBC (*N* = 43, 15% missing). Five iterations produced five imputed datasets. Imputed scores were pooled and combined with the non-imputed dataset. A sensitivity analysis was conducted on the raw and imputed datasets with no differences in conclusions.

## Results

### Patient characteristics

**Figure 1.**
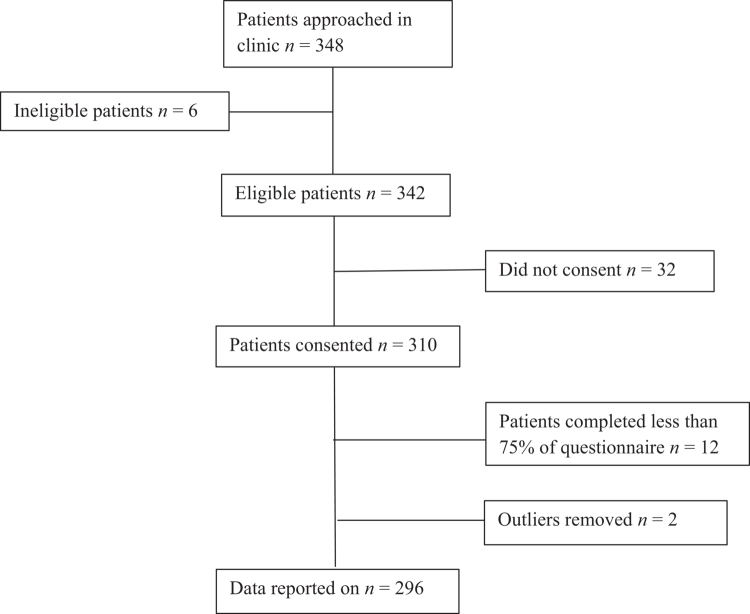
Participant recruitment flow diagram.

*N* = 348 KTR were approached over 12 months. Six (1.7%) patients were ineligible as they did not speak English. Out of the 342 eligible patients, 310 (90.6%) consented. Twelve patients (3.8%) completed less than 75% of the questionnaire and were excluded from analyses. Two outliers were removed. The final study sample included 296 KTR (see Figure 1).

A summary of demographic and clinical factors is presented in Table 1. The mean age was 50.07 years (SD = 14.29), with males representing just over half of the sample (*N* = 158, 53%). There was an even proportion of White British patients (*N* = 153, 52%) and patients from other ethnic backgrounds (*N* = 143, 48%). One third (*N* = 99, 33%) lived in areas representing the second most deprived quintile of the population in England.

**Table 1.** Demographic and clinical characteristics of the total sample and adherence groups.

		Total non-adherence (MARS ≤ 24)		Intentional non-adherence (MARS ≤ 19)		Unintentional non-adherence (MARS ≤ 4)	
Characteristic	Total sample (*N* = 296)	Adherent (*n* = 126)	Non-adherent (*n* = 170)	Adherent (*n* = 255)	Non-adherent (*n* = 41)	Adherent (*n* = 135)	Non-adherent (*n* = 161)
Age, mean (SD)	50.07 (14.29)	51.45 (14.01)	49.05 (14.45)	50.75 (14.13)*	45.87 (14.72)*	51.21 (13.88)	49.11 (14.59)
Sex *n* (%)							
Male	158 (53)	65 (52)	93 (55)	137 (54)	21 (51)	70 (52)	88 (55)
Female	138 (47)	61 (48)	77 (45)	118 (46)	20 (49)	65 (48)	73 (45)
Ethnicity *n* (%)							
White British	153 (52)	65 (52)	88 (52)	134 (53)	19 (46)	71 (53)	82 (51)
Other ethnic background	143 (48)	61 (48)	82 (48)	121 (47)	22 (54)	64 (47)	79 (49)
IMD quintile *n* (%)							
First (most deprived)	36 (12)	16 (13)	20 (12)	35 (14)	1 (2)	16 (12)	20 (12)
Second	99 (33)	40 (32)	59 (35)	81 (32)	18(44)	42 (31)	57 (35)
Third	62 (21)	29 (23)	33 (19)	54 (21)	8 (20)	31 (23)	31 (19)
Fourth	56 (19)	25 (20)	31 (18)	49 (19)	7 (17)	28 (21)	28 (18)
Fifth (least deprived)	43 (15)	16 (12)	27 (16)	36 (14)	7 (17)	18 (13)	25 (16)
Donor type *n* (%)							
Living	119 (40)	48 (38)	71 (42)	100 (39)	19 (46)	50 (37)	69 (43)
Deceased	177 (60)	78 (62)	99 (58)	155 (61)	22 (54)	85 (63)	92 (57)
Time since transplantation (years) ~	5.70 (11.5)	4.40 (10.9)*	6.10 (11.0)*	5.70 (11.77)	5.50 (10.6)	4.40 (10.05)**	6.70 (11.21)**
Number of kidney transplants *n* (%)							
One	237 (80)	99 (79)	138 (81)	205 (80)	32 (78)	103 (76)	134 (83)
Two or more	59 (20)	27 (21)	32 (19)	50 (20)	9 (22)	32 (24)	27 (17)
Number of prescribed immunosuppressive therapies *n* (%)							
One	7 (2)	4 (2)	3 (2)	6 (2)	1 (2)	5 (3)	2 (1)
Two	85 (29)	38 (31)	47 (27)	77 (30)	8 (20)	40 (30)	45 (28)
Three	204 (69)	84 (67)	120 (71)	172 (68)	32 (78)	90 (67)	114 (71)
Comorbidities (Charlson Comorbidity Index Score) ~	4 (2)	4 (2)	4 (2)	4 (2)	4 (2)	4 (2)	4 (2)
Pill burden (total number of tablets per day) ~	14 (8)	16 (10)⁺	13 (7)⁺	14 (8)	14 (11)	16 (10)⁺	13 (7)⁺
Depression or anxiety diagnosis *n* (%)							
Yes	79 (27)	20 (16)⁺	59 (35)⁺	61 (24)**	18 (44)**	25 (19)**	54 (34)**
No	217 (73)	106 (84)⁺	111 (65)⁺	194 (76)^**^	23 (56)**	110 (81)	107 (66)**
eGFR (ml/min/1.73 m²), mean (SD)	44.92 (22.63)	44.47 (23.90)	45.25 (21.69)	44.89 (21.80)	45.10 (27.51)	45.13 (24.12)	44.74 (21.36)
Serum creatinine (μmol/L) ~	135.50 (84)	139.50 (92)	133.00 (78)	136.00 (79)	135.00 (105)	140.00 (85)	133 (81.5)

Note: IMD = Index of Multiple Deprivation, ~Median and Interquartile Range.

* Significant group difference at *p *< .05.

** Significant group difference at *p *< .01.

^+^ Significant group difference at *p* < .001.

Reference values for clinical parameters: normal eGFR ≥90 mL/min/1.73 m², serum creatinine 45–104 µmol/L (Varies by age/sex).

Intentional and unintentional non-adherence are not mutually exclusive; participants could report both.

Most patients were living with their first transplant (*N* = 237, 80%). The median length of time since transplantation was 5.7 years (minimum = one month, maximum = 44.7 years). *N* = 177 KTR (60%) received their transplant from a deceased donor. *N* = 204 (69%) patients were prescribed triple immunosuppressive therapy. The mean eGFR was 44.92 ml/min/1.73 m² (SD = 22.63) and median serum creatinine was 135.50 (μmol/L) (IQR = 84).

### Rates of non-adherence

The mean overall MARS score was 23.96 (SD = 1.41). *N* = 170 (57%) participants were classified as non-adherent overall. Intentional non-adherence was reported by *N* = 41 (14%), with a mean score of 19.65 (SD = 1.08). *N* = 161 (54%) participants reported unintentional non-adherence, with a mean score of 4.32 (SD = 0.72).

There was a trend towards those who showed non-adherence (total non-adherence score) to have a greater time since transplantation (U = 8914.5, *p* = .014 (Table 1), however this did not reach significance when controlling for multiple testing. A lower pill burden (U = 7946.5, *p *< .001) and a diagnosis of depression or anxiety (χ2(1,296) = 13.118, *p *< .001) were significantly associated with total non-adherence.

There was a trend towards those who were intentionally non-adherent to be younger, (t(294) = 2.04, *p* = .043) and have a diagnosis of depression or anxiety (χ2(1,296) = 7.207, *p* = .007) but again, these trends did not reach significance when controlling for multiple testing (Table 1).

Unintentional non-adherence was more likely in participants with a clinical diagnosis of depression or anxiety (χ2(1,296) = 8.469, *p* = .004) and longer time since transplantation (U = 8837.5, *p* = .006), though these did not reach significance after correction. Lower pill burden was significantly associated with unintentional non-adherence (U = 8154, *p *< .001) (Table 1).

### Psychological correlates of total, intentional and unintentional non-adherence

Means (standard deviations) of psychological variables and correlations between total, intentional and unintentional MARS non-adherence scores are presented in Table 2.

**Table 2.** Summary statistics and correlations between psychological and behavioural variables and total, intentional and unintentional non-adherence.

	Possible range	Mean (SD)	Correlation with total MARS scores	Correlation with intentional MARS scores	Correlation with unintentional MARS scores
Distress (PHQ−4)	0−12	2.27 (2.55)	−0.128*	−0.037	−0.195***
Depression/anxiety diagnosis *n*, (%)					
Yes		79 (27)	t(294) = 3.75,	t(294) = 2.49,	t(294) = 4.15,
No		217 (73)	*p *< .001	*p* = .015	*p *< .001
Work & social adjustment (WSAS)	0−40	12.61 (11.79)	−0.079	−0.116*	0.018
**Illness/transplant perceptions**					
Consequences (CKD)	0−10	4.55 (2.81)	−0.035	−0.026	−0.030
Consequences (Tx)	0−10	6.57 (3.24)	0.065	0.080	0.007
Timeline (CKD)	0−10	8.21 (2.74)	−0.025	0.039	−0.106
Personal control (CKD)	0−10	5.47 (2.65)	0.132*	0.122*	0.076
Personal control (Tx)	0−10	5.76 (2.86)	0.133*	0.120*	0.081
Treatment control (CKD)	0−10	8.05 (2.07)	0.090	0.122*	-0.006
Treatment control (Tx)	0−10	8.06 (2.01)	0.034	0.019	0.039
Identity (CKD)	0−10	4.42 (2.70)	−0.088	−0.065	−0.074
Concern (CKD)	0−10	5.79 (2.90)	−0.080	−0.004	−0.152^**^
Concern (Tx)	0−10	6.43 (3.12)	−0.075	−0.038	−0.090
Coherence (CKD)	0−10	8.28 (1.81)	0.182*	0.105	0.201***
Coherence (Tx)	0−10	7.52 (2.56)	0.086	0.051	0.093
Emotional impact (CKD)	0−10	4.69 (2.93)	−0.134*	−0.095	−0.121*
Emotional impact (Tx)	0−10	4.65 (3.41)	−0.102	−0.050	−0.126*
**Medication beliefs**					
BMQ-Specific necessity	5−25	21.43 (3.54)	0.018	0.046	−0.033
BMQ-Specific concerns	5−25	12.65 (3.84)	−0.124*	−0.069	−0.139*
BMQ-Specific *N*-C differential	−20−20	8.78 (5.42)	0.100	0.079	0.077
Perceived behavioural control surrounding medication taking	4−28	25.86 (3.00)	0.314***	0.240***	0.255***

Note: PHQ−4 = Patient Health Questionnaire; WSAS = Work and Social Adjustment Scale; CKD = chronic kidney disease perception; Tx = transplant graft-related perception.

* *p *< .05.

** *p *< .01.

*** *p *≤ .001.

Distress was low and significantly negatively correlated with total (*r* = −.128) and unintentional (*r* = −.195) non-adherence. Participants with a depression or anxiety diagnosis reported significantly poorer adherence across all outcomes. Impact of CKD on work and social adjustment was low and significantly negatively correlated with intentional non-adherence.

Illness perception means were generally mid-range with timeline, treatment control and coherence being higher. Means for directly comparable CKD and graft-related perceptions were generally similar, though graft failure was perceived as having greater consequences, being more concerning, and less well understood.

Many CSM illness perceptions were not significantly correlated with adherence outcomes, and those that were showed small effects. Unintentional non-adherence tended to correlate significantly with negative perceptions, including concern about CKD (*r* = −.152), ISM (*r* = −.139), and emotional impact of CKD (*r* = −.121) and risk of graft failure (*r* = −1.26). Conversely, intentional non-adherence was significantly correlated only with perceived control over CKD (*r* = .122), graft failure (r = .120), and treatment (*r* = .122). Greater understanding of CKD, but not of the risk of graft failure, was significantly correlated with total (*r* = .182) and unintentional (*r* = .201) non-adherence.

Perceived behavioural control over medication taking measured by the TPB had small-moderate significant correlations with total (*r* = .314), intentional (*r *= .240) and unintentional (*r* = .255) non-adherence.

### Multivariate logistic regression: factors associated with total non-adherence (MARS ≤ 24)

A hierarchical logistic regression model included all variables that showed significant bivariate associations with total non-adherence (Table 3). Step 1 included demographic and clinical factors, i.e. pill burden. Depression–anxiety diagnosis was added into Step 2. CSM components in Step 3 were perceived personal control over CKD, personal control over graft loss, CKD coherence, emotional impact of CKD and ISM concerns. CSM and PBC were tested separately, followed by a combined model.

**Table 3.** Hierarchical binary logistic regression: factors associated with total non-adherence (MARS ≤ 24 = 1).

							Step 3								
Variables	Step 1			Step 2			CSM			PBC			CSM and PBC		
	OR	95% CI	Sig	OR	95% CI	Sig	OR	95% CI	Sig	OR	95% CI	Sig	OR	95% CI	Sig
Pill burden	0.95	0.92−0.98	.001	0.93	0.90−0.97	<.001	0.94	0.90−0.97	<.001	0.93	0.90−0.96	<.001	0.93	0.90−0.97	<.001
Depression–anxiety diagnosis				3.58	1.95−6.59	<.001	3.28	1.72−6.26	<.001	3.38	1.81−6.31	<.001	3.30	1.71−6.36	<.001
Personal control (CKD)							0.97	0.87–1.08	.545				0.98	0.88−1.09	.641
Personal control (Tx)							0.94	0.85−1.04	.210				0.94	0.85−1.03	.174
Coherence (CKD)							0.85	0.73−0.99	.042				0.90	0.77−1.06	.195
Emotional impact (CKD)							0.99	0.89−1.10	.788				0.98	0.88−1.08	.674
BMQ Concerns							1.01	0.94−1.09	.829				1.00	0.93−1.07	.930
PBC										0.85	0.77−0.94	.002	0.87	0.78−0.97	.010
	−2LL: 392.280 R²:.051 χ2: 11.498 *p *< .001			−2LL: 373.388 R²:.131 ∆ R²:.08 Step χ2: 18.892 Model χ2: 30.390 ∆ Model χ2: 18.892 Step *p *< .001 Model *p *< .001			−2LL: 363.917 R²: .169 ∆ R²:.038 Step χ2: 9.471 Model χ2: 39.861 ∆ Model χ2: 9.471 Step *p* = .092 Model sig *p *< .001			−2LL: 362.379 R²:.175 ∆ R²:.044 Step χ2: 11.009 Model χ2: 41.399 ∆ Model χ2: 11.009 Step *p *< .001 Model sig *p *< .001			−2LL: 356.625 R²:.198 ∆ R²:.067 Step χ2: 16.764 Model χ2: 47.154 ∆ Model χ2: 16.764 Step *p* = .010 Model sig *p *< .001		

Note: CKD = chronic kidney disease perception. Tx = transplant graft-related perception.

The model combining the CSM and PBC explained the most variance in total non-adherence (Nagelkerke *R*² = 19.8%) (Table 3). In this model, pill burden in Step 1 explained 5.1% of the variance, χ2 (1) = 11.498, *p *< .001, *R*² = 5.1%. A higher number of pills (OR = 0.95, 95% CI = 0.92−0.98) was associated with decreased odds of total non-adherence. The addition of depression–anxiety diagnosis in Step 2 significantly improved model fit and explained a further 8% of the variance (χ2 (2) = 30.390, *p *< .001, *R*² = 13.1%).

Adding the CSM variables and PBC significantly improved the model fit and explained a further 6.7% of the variance, (χ2 (8) = 47.154, *p *< .001, *R*² = 19.8%). Higher levels of PBC (OR = 0.87, 95% CI = 0.78−0.97) were associated with decreased odds of non-adherence. No CSM variables were significant in the final model. Pill burden (OR = 0.93, 95% CI = 0.90−0.97) and depression/anxiety diagnosis (OR = 3.30, 95% CI = 1.71−6.36) remained significant. The model correctly classified 78.2% of non-adherent participants.

### Multivariate logistic regression: factors associated with intentional non-adherence (MARS ≤ 19)

Similar hierarchical binary logistic regressions were conducted with intentional non-adherence as the outcome. No clinical or demographic variables were significantly associated. Therefore, depression/anxiety diagnosis and WSAS were entered into Step 1 (Table 4). CSM components in Step 2 included perceived personal control over CKD, personal control over graft loss and CKD treatment control. CSM components and PBC were tested separately, followed by a combined model.

**Table 4.** Hierarchical binary logistic regression: factors associated with intentional non-adherence (MARS ≤ 19 = 1).

				Step 2								
Variables	Step 1			CSM			PBC			CSM and PBC		
	OR	95% CI	Sig	OR	95% CI	Sig	OR	95% CI	Sig	OR	95% CI	Sig
Depression/anxiety diagnosis	2.31	1.13−4.74	.022	2.13	1.02−4.47	.045	2.07	0.98−4.36	.056	2.01	0.93−4.33	.076
Work & social adjustment	1.01	0.98−1.04	.531	1.00	0.97−1.03	.927	1.00	0.97−1.03	.982	0.99	0.96−1.03	.689
Personal control (CKD)				0.89	0.76−1.04	.137				0.87	0.74−1.03	.102
Personal control (Tx)				0.94	0.82−1.08	.371				0.95	0.83−1.10	.500
Treatment control (CKD)				0.93	0.80−1.09	.387				0.96	0.81−1.13	.595
PBC							0.84	0.76−0.93	<.001	0.85	0.77−0.94	.001
	−2LL: 231.118 R²:.042 χ2: 7.018 *p* = .030			−2LL: 222.690 R²:.092 ∆ R²: .05 Step χ2: 8.428 Model χ2: 15.446 ∆ Model χ2: 8.428 Step *p* = .038 Model *p* = .009			−2LL: 218.778 R²:.115 ∆ R²:.073 Step χ2: 12.340 Model χ2: 19.357 ∆ Model χ2: 12.339 Step *p *< .001 Model *p *< .001			−2LL: 211.637 R²:.155 ∆ R²:.113 Step χ2: 19.481 Model χ2: 26.499 ∆ Model χ2: 19.481 Step *p *< .001 Model *p *< .001		

Note: CKD = chronic kidney disease perception. Tx = transplant graft-related perception.

The model including a combination of CSM variables and PBC explained the most variance in intentional non-adherence (Nagelkerke *R*² = 15.5%) (Table 4). Depression/anxiety diagnosis and WSAS in Step 1 explained 4.2% of the variance χ2 (2) = 7.018, *p* = .030, *R*² = 4.2%. Adding the CSM variables and PBC significantly improved model fit and explained a further 11.3% of variance, (χ2 (6) = 26.499, *p *< .001, *R*² = 15.5%). Higher levels of PBC (OR = 0.85, 95% CI = 0.77−0.94) were associated with decreased odds of intentional non-adherence. No CSM variables were significant in the final model. The model correctly classified 10% of intentionally non-adherent participants.

### Multivariate logistic regression: factors associated with unintentional non-adherence

Hierarchical binary logistic regressions were also conducted for unintentional non-adherence (Table 5). Step 1 included demographic and clinical factors, i.e. pill burden. Depression/anxiety diagnosis was added into Step 2. In terms of variables from the CSM, unintentional non-adherence was associated with CKD concern, CKD coherence, emotional impact of CKD, emotional impact of graft loss and ISM concerns. These were added into Step 3. Separate logistic regressions tested components of the CSM, PBC, and the combined model.

**Table 5.** Hierarchical binary logistic regression: factors associated with unintentional non-adherence (MARS ≤ 4 = 1).

							Step 3								
Variables	Step 1			Step 2			CSM			PBC			CSM and PBC		
	OR	95% CI	Sig	OR	95% CI	Sig	OR	95% CI	Sig	OR	95% CI	Sig	OR	95% CI	Sig
Pill burden	0.95	0.92−0.98	<.001	0.93	0.90−0.97	<.001	0.93	0.90−0.97	<.001	0.93	0.90−0.96	<.001	0.93	0.89−0.96	<.001
Depression–anxiety diagnosis				2.76	1.55−4.92	<.001	2.39	1.30−4.42	.005	2.57	1.41−4.66	.002	2.40	1.29−4.49	.006
Concern (CKD)							1.07	0.95−1.21	.296				1.10	0.97−1.25	.141
Coherence (CKD)							0.84	0.73−0.98	.023				0.90	0.78−1.05	.177
Emotional (CKD)							0.96	0.84−1.10	.492				0.92	0.80−1.05	.215
Emotional (Tx)							1.03	0.94−1.13	.555				1.04	0.95−1.15	.388
BMQ concerns							1.02	0.95−1.10	.565				0.98	0.93−1.08	.977
PBC										0.83	0.75−0.92	<.001	0.84	0.75−0.93	.001
	−2LL: 395.959 R²:.054 χ2: 12.097 *p *< .001			−2LL: 383.122 R²:.108 ∆ R²:.054 Step χ2: 12.838 Model χ2: 24.935 ∆ Model χ2: 12.838 Step *p *< .001 Model *p *< .001			−2LL: 373.699 R²:.146 ∆ R²:.038 Step χ2: 9.422 Model χ2: 34.357 ∆ Model χ2: 9.422 Step *p* = .093 Model *p *< .001			−2LL: 367.539 R²: .171 ∆ R²:.063 Step χ2: 15.582 Model χ2: 40.517 ∆ Model χ2: 15.582 Step *p *< .001 Model *p *< .001			−2LL: 361.788 R²: .193 ∆ R²:.085 Step χ2: 21.334 Model χ2: 46.269 ∆ Model χ2: 21.334 Step *p* = .002 Model *p *< .001		

Note: CKD = chronic kidney disease perception. Tx = transplant graft-related perception.

The model combining CSM variables and PBC explained the most variance in unintentional non-adherence (Nagelkerke *R*² = 19.3%) (Table 5). Pill burden in Step 1 explained 5.4% of the variance, χ2 (1) = 12.097, *p *< .001, *R*² = 5.4%. A higher number of pills (OR = 0.95, 95% CI = 0.92−0.98) was associated with decreased odds of unintentional non-adherence. Adding depression–anxiety diagnosis in Step 2 significantly improved model fit, explaining an additional 5.4% of the variance (χ2 (2) = 24.935, *p *< .001, *R*² = 10.8%).

Adding CSM variables and PBC in Step 3 significantly improved model fit and explained a further 8.5% of variance (χ2 (8) = 46.269, *p *< .001, *R*² = 19.3%). Higher levels of PBC (OR = 0.84, 95% CI = 0.75−0.93) were associated with decreased odds of unintentional non-adherence. No CSM variables were significant in the final model. Pill burden (OR = 0.93, 95% CI = 0.89−0.96) and a diagnosis of depression–anxiety (OR = 2.40, 95% CI = 1.29−4.49) remained significant in Step 3. The model correctly classified 68.9% of unintentionally non-adherent participants.

## Discussion

This was the first study to examine both graft-specific as well as broader CKD perceptions, in conjunction with beliefs about ISM, and PBC around taking ISM in relation to total, intentional and unintentional non-adherence in KTRs. Results identified unique psychological correlates for intentional and unintentional non-adherence, highlighting the distinction and the need for targeted interventions to address medication non-adherence in this patient cohort. While perceptions around graft loss were more negative, CKD-related perceptions were more strongly related to adherence outcomes. This suggests that addressing unfavourable perceptions around CKD, particularly coherence and personal control, may be effective intervention targets. Supporting our hypothesis, the addition of PBC to CSM variables increased the variance explained in all adherence behaviours.

Over half of participants (57%) reported any indication of non-adherence, consistent with documented rates among KTRs (Cossart et al., 2019; Griva et al., 2012; Lennerling & Forsberg, 2012). As hypothesised, and in line with past research (Griva et al., 2018), unintentional non-adherence (54%) was reported more frequently than intentional non-adherence (14%). It is unclear whether this reflects genuinely more frequent forgetting or greater social desirability in reporting. As self-report measures underestimate non-adherence (Chan et al., 2020), the high rates observed here reinforce the significance of the problem, particularly given the sevenfold increased risk of graft failure among non-adherent patients (Butler et al., 2004). The unique psychological correlates of intentional and unintentional non-adherence further support the behavioural distinction.

Most demographic and clinical characteristics were not associated with adherence, aligning with inconsistent findings in KTR research (Gokoel et al., 2020). Contrary to past research (Belaiche et al., 2017) and not in support of our hypothesis, greater pill burden was associated with reduced odds of total and unintentional non-adherence. One explanation is that those with fewer pills may perceive their illness as less severe and the consequences of non-adherence to be lower. Another possibility is that a high pill burden may lead patients to develop better organisational strategies for their complex regimens. This should be explored in future research to identify successful techniques. Tools such as dosette boxes could help reduce forgetting (Conn et al., 2015), and pharmacists, often involved in overseeing complex regimens, are well-positioned to implement similar interventions.

The association between younger age and intentional non-adherence may reflect lower perceived consequences of non-adherence among younger patients, who may also experience greater lifestyle disruption. In contrast, the association between longer time since transplant and unintentional non-adherence may reflect a gradual shift in focus away from the transplant as everyday demands take precedence, increasing the likelihood of accidental lapses.

In our sample, CKD impact on work and functioning, and distress, were low, likely reflecting clinical stability further out from transplantation or adaptation to CKD over time. This may underestimate their true contribution to adherence, as these factors could be more influential for newly transplanted or less well-adapted patients. Nevertheless, a clinical diagnosis of depression or anxiety (affecting 27% of the sample) was associated with all adherence behaviours. Although the cross-sectional design limits causal conclusions, this finding merits further investigation into the role of mental health in post-transplant adherence.

Participants strongly believed in the importance of taking their ISM, regardless of their adherence, a finding consistent with previous research in KTRs (Cossart et al., 2019; Lennerling & Forsberg., 2012; Massey et al., 2013). Concerns about ISM were generally low, also consistent with prior research (Cossart et al., 2019; Lennerling & Forsberg, 2012). Unexpectedly, greater concerns were associated with total and unintentional non-adherence, but not with intentional non-adherence, possibly indicating reduced motivation and avoidance behaviours. This suggests a more subconscious process whereby concerns may increase the likelihood of forgetting, acknowledged by Horne et al. (2013). However, concerns were not significant in multivariate models, supporting previous findings (Lennerling & Forsberg, 2012).

While directly comparable CKD and graft-related perceptions (e.g. CKD-related coherence versus graft-related coherence) were generally similar, graft failure was perceived as more consequential, more concerning, and less well understood. However, adherence behaviours were more strongly correlated with perceptions of CKD, suggesting that the more familiar and enduring health threat of CKD may have a greater influence on behaviour than the perceived risk of graft loss. This supports the concept of a dual health threat, where KTRs perceive both the ongoing risk of CKD progression and graft failure as distinct threats. Clinically, presenting ISM adherence not only as a means of protecting the graft but as an essential part of CKD management may be beneficial for adherence. Future research could explore how these perceptions interact and change over time.

The model combining CSM variables and perceived behavioural control (PBC) explained 19.8% of total non-adherence, 15.5% of intentional non-adherence and 19.3% of unintentional non-adherence, highlighting the role of illness perceptions alongside PBC. Perceptions associated with unintentional non-adherence were largely emotionally driven, potentially reflecting avoidant ‘forgetting’ in emotionally affected KTRs. In contrast, intentional non-adherence was primarily associated with control-related perceptions: personal control over CKD, graft loss, and CKD treatment control. This aligns conceptually and reinforces the relevance of control-related constructs. While the CSM emphasises beliefs about illness and treatment control, the TPB focuses on actual behavioural control; integrating both may better explain behaviour in specific contexts. Our findings support the predictive role of PBC for intentional non-adherence, consistent with previous findings (Moon et al., 2017). Overall, the distinct contributions of variables to intentional and unintentional non-adherence reinforce the need to explore these behaviours separately, as they hold distinct intervention targets.

Improving PBC around taking ISM may be beneficial for both adherence behaviours. Implementation intentions—structured ‘if-then’ plans, ‘I intend to do X at time Y in location Z’—have increased adherence in various contexts, such as cervical cancer screening uptake (Sheeran & Orbell, 2000) and medication adherence in stroke survivors (O’Carroll et al., 2013). Combining implementation intentions with behaviour change techniques to modify negative illness perceptions, such as improving perceived understanding of CKD, may help reduce forgetfulness. Given that higher pill burden was associated with better adherence in our sample, possibly reflecting stronger organisational strategies, such interventions may be particularly effective when building on existing engagement with general medication taking.

Although CSM variables did not remain significant in the combined models, their inclusion alongside PBC improved explanatory power, suggesting value in their integration. However, the variance explained by the models was modest, suggesting other factors such as social support, health literacy, or the patient-provider relationship, are also important (Corr et al., 2024). Future longitudinal research is needed to explore how these factors, and the ways in which CSM variables and PBC interact and change over time, influence adherence. In addition, qualitative research could provide insight into how patients distinguish between the dual threats of CKD and graft-perceptions, and how the different perceptions relate to intentional versus unintentional non-adherence.

This study has several limitations. Self-reported adherence may be subject to social desirability bias; however, MARS items are phrased non-judgmentally to reduce this effect and are known to underestimate non-adherence (Chan et al., 2020). Additionally, MARS total and unintentional scores have previously shown significant correlations with clinical proxies such as sub-target serum immunosuppressive concentrations, supporting its validity (Griva et al., 2012). Another limitation is that unintentional non-adherence was operationalised using a single MARS item assessing forgetfulness, which does not capture other unintentional behaviours (e.g. misunderstanding instructions). Future research should incorporate measures to reflect this.

The cross-sectional design of the study limits causal inference. Adherence is conceptualised by the CSM as dynamic and has been shown to decrease over time post-transplant (Couzi et al., 2013; Nevins et al., 2014). While longitudinal studies have examined illness perceptions and beliefs in KTRs (Massey et al., 2013, 2015), future research should extend this work by exploring the predictive value of PBC on separate adherence behaviours over time.

Despite these limitations, the study has several strengths. It was conducted at the largest transplant centre in the UK, serving a demographically diverse population. The demographic profile of the sample mirrors national KTR data (NHS Blood and Transplant, 2024), supporting the generalisability of findings to the wider UK kidney transplant population. Additionally, the robust sample size, adequate power and high consent rate (90%) increase the reliability of results and reduces selection bias.

In conclusion, this study highlights the significance of non-adherence in KTRs, particularly the high rate of unintentional non-adherence. Findings reinforce the distinction between intentional and unintentional non-adherence, and their unique associated factors, which should inform intervention development. Enhancing PBC around medication taking has been established as a promising target to potentially improve both types of adherence. Future longitudinal research is needed to identify the most predictive variables for ISM adherence in KTRs to inform effective interventions.

## Data Availability

The data that support the findings of this study are available from the corresponding author upon reasonable request.
